# Unexpected Strikes: Sjögren Syndrome Triggering Multiple Evolving Cerebral Strokes in a Young Patient

**DOI:** 10.7759/cureus.65905

**Published:** 2024-07-31

**Authors:** Priti Pawar, Deepika Kanyal, Abhinav Kadam, Shweta Khare

**Affiliations:** 1 Department of Hospital Administration, Datta Meghe Institute of Higher Education and Research, Wardha, IND; 2 Department of Medicine, Jawaharlal Nehru Medical College, Datta Meghe Institute of Higher Education and Research, Wardha, IND

**Keywords:** cerebrovascular event, central nervous system, seizures, vasculitis, autoimmune

## Abstract

Neurological complications are observed less frequently with primary Sjögren syndrome (SS). The central nervous system (CNS) has seldom been shown to exhibit symptoms of SS, making the diagnosis of SS with neurological involvement difficult. We present a rare case scenario in which a young 23-year-old male presenting with an acute history of fever, headache, vomiting, altered sensorium, and seizures was admitted and diagnosed as a sub-acute infarct in the right frontal-parietal-temporal lobes on a computed tomography (CT) scan. Upon further examination, laboratory investigations were suggestive of viral encephalitis. The patient was treated accordingly with antiviral drugs, and the patient improved. The patient took "discharge against medical advice" after 12 days, only to return to the hospital with similar complaints within 15 days. Magnetic resonance imaging (MRI) was done, which suggested an acute evolving infarct in the right frontal and parietal lobe, and further evaluation yielded a diagnosis of SS. The patient was treated with high-dose steroids for seven days. A repeat MRI showed new acute infarcts with dilatation of the ventricular system with periventricular ooze. The patient could not be revived and succumbed after one week of steroid therapy.

## Introduction

Exocrine glands are affected majorly by the multisystemic autoimmune disease known as Sjögren syndrome (SS). Although it mainly affects the lacrimal and salivary glands, extra-glandular symptoms have been observed in 33% of the patients [1,2]. Seventy percent of patients experience extra-glandular problems, frequently affecting the skin, lungs, joints, and peripheral nervous system (PNS). In rare cases, the central nervous system (CNS) may be involved [3]. The pathological processes behind the dysfunction of the neurological system remain unclear. However, various theories have been proposed, including the invasion of inflammatory cells, autoantibody-mediated vascular damage, and ischemia resulting from small-vessel vasculitis.

Small arteries are more commonly affected by cerebral vasculitis linked to Sjögren's disease than medium or large arteries. Although ischemic strokes account for the bulk of known cases, none of them showed signs of haemorrhagic change, and none of them occurred in such extensive cerebral vascular areas. Before evaluating a particular neurological involvement in individuals with SS who exhibit neurological symptoms, other possible diagnoses must be ruled out, such as cryoglobulinaemic vasculitis and related autoimmune disorders (systemic lupus erythematosus (SLE), multiple sclerosis, etc.).

## Case presentation

**Figure 1:**
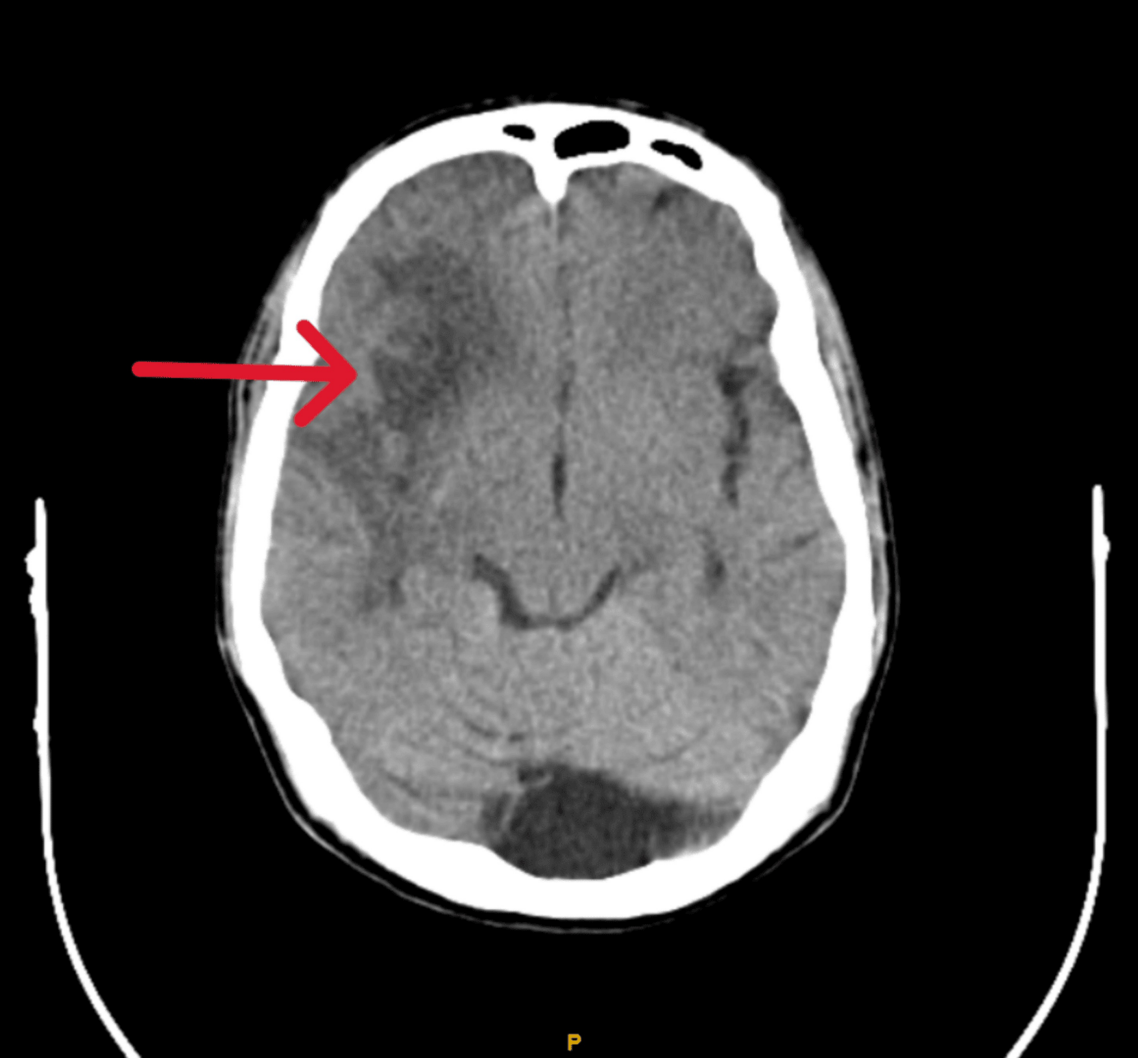
CT scan of the brain showing sub-acute infarct of the right frontal-parietal-temporal regions involving the right ganglion-capsular regions and right corona radiata (red arrow). CT: computed tomography

A 23-year-old male came to the emergency department with chief complaints of fever (on and off) for one month, headache, vomiting (2-3 episodes), weakness of the left upper and lower limb, seizures (2-3 episodes), and altered sensorium for one day. There were no other complaints. The patient was not a known case of any co-morbidity and was not taking any medications. On examination, the patient's pulse rate was 94 beats per minute, blood pressure was 130/80 mmHg, and oxygen saturation was 98% on room air. He was lying comfortably in bed and showed no signs of distress. There were no pallor, icterus, clubbing, cyanosis, or lymphadenopathy. There were no evident dermatological lesions on examination. Cardiovascular examination was suggestive of insignificant findings. The respiratory system was found to show normal findings. Upon neurological examination, the patient had an altered sensorium. Neck stiffness was present, and Kernig's sign was positive. Power was found in the lower limb as 5/5 on the right side and 3/5 on the left side, whereas in the upper limb as 5/5 on the right side and 2/5 on the left side. Pyramidal signs were positive on the left side of the body. Higher mental functions, sensory examination, and cerebellar signs could not be examined due to altered sensorium. A computed tomography (CT) scan of the brain was done, which showed sub-acute infarct of the right frontal-parietal-temporal regions involving the right ganglion-capsular regions and right corona radiata (Figure 1).

A complete laboratory investigation of the patient was requested (shown in Table 1). It suggested mild iron deficiency anaemia, normal kidney and liver function tests, normal thyroid profile, and other insignificant findings. Inflammatory markers were found to be raised (erythrocyte sedimentation rate (ESR): 84 millimeter/hour, normal range: <15 millimeter/hour; C-reactive protein (CRP): 36 milligram/deciliter, normal range: <0.3 milligram/deciliter). An antinuclear antibody using the enzyme-linked immunosorbent assay (ELISA) method was investigated, and it was found to be 2.525 (normal reference range: >1.1 considered positive). A lumbar puncture was planned after a fundus review. Cerebrospinal fluid (CSF) analysis was done, which was suggestive of the findings, as shown in Table 2. Tuberculosis-polymerase chain reaction (TB-PCR) and herpes simplex virus-PCR (HSV-PCR) were also sent to enquire about tubercular meningitis, which came back negative. Anti-ds-DNA by ELISA was done which was found to be 11.65 IU/ml (normal range: <30 IU/ml). Anticardiolipin antibody (ACA) and beta-2-glycoprotein I (B2GPI) antibody were done which both came as negative. 

**Table 1:** Laboratory findings of the patient with their normal reference ranges on admission. g/dl: gram per deciliter; micron: micrometer; pg: picogram; cumm: cubic millimeter; fL: femtoliter; mg/dl: milligram/deciliter; pg/dl: picogram per deciliter; ng/dl: nanogram per deciliter; mEq/L: milliequivalent/liter; IU/L: international units/liter; U/L: units/liter; MCHC: mean corpuscular hemoglobin concentration; MCV: mean corpuscular volume; MCH: mean corpuscular hemoglobin; RBC: red blood cell; WBC: white blood cell; RDW: red cell distribution width; HbA1C: glycated hemoglobin; SGOT: serum glutamic oxaloacetic transaminase; SGPT: serum glutamic pyruvic transaminase; HIV: human immunodeficiency virus; HBsAg: hepatitis B surface antigen; HCV: hepatitis C virus

Laboratory parameter	Results	Normal values
Hemoglobin	11.2 Gm/dl	11-14 Gm/dl
MCHC	32.5 Gm/dl	32-36 Gm/dl
MCV	64.8 micron	79-92 micron
MCH	21.7 pg	27-31 pg
Total RBC count	5.39 x 10^6 ^cells/cumm	2.50-5.50 x 10^6 ^cells/cumm
Total WBC count	7300 cells/cumm	4000-11000 cells/cumm
Total platelet count	3.17 x 10^6 ^cells/cumm	1.50-4.50 x 10^6 ^cells/cumm
Hematocrit	34.5%	40-54%
Monocyte	4%	2-8%
Granulocyte	75%	40-60%
RDW	18.8 fL	12.2-16.1 fL
Eosinophil	1%	1-4%
Basophil	0%	<1%
Random blood sugar level	96 mg/dl	70-140 mg/dl
Serum ferritin	22.8 ng/ml	17.9-466 ng/ml
Free T3	3.51 pg/ml	2.77-5.27 pg/ml
Free T4	1.63 ng/ml	0.78-2.19 ng/dl
Thyroid stimulating hormone	0.457 mIU/ml	0.465-4.68 mIU/ml
Urea	34 mg/dl	6.24 mg/dl
Creatinine	1.0 mg/dl	0.59-1.04 mg/dl
Sodium	132 mEq/l	135-145 mEq/l
Potassium	4.6 mEq/l	3.5-5.1 mEq/l
HbA1C	4.7%	<6%
Alkaline phosphate	98 IU/L	75-124 IU/L
SGOT	21 IU/L	8-45 IU/L
SGPT	17 IU/L	7-56 IU/L
Total protein	6.7 g/dl	6.0-8.3 g/dl
Albumin	3.6 g/dl	3.4-5.4 g/dl
Total bilirubin	0.5 mg/dl	0.1-1.0 mg/dl
Conjugated bilirubin	0.2 mg/dl	0.1-0.4 mg/dl
Unconjugated bilirubin	0.3 mg/dl	0.2-0.6 mg/dl
Serum homocysteine	6.62 mMol/l	6.6-14.8 mMol/l
Serum iron	35 microgm/dl	49-181 microgm/dl
Creatinine-urine	53.5 mg/dl	20-320 mg/dl
Protein-urine	28 mg/dl	0-14 mg/dl
Urine protein/creatinine ratio	0.52 mg/mg	0.15-0.50 mg/mg
Total cholesterol	115 mg/dl	<200 mg/dl
Triglycerides	89 mg/dl	<150 mg/dl
High-density lipoprotein	43 mg/dl	40-60 mg/dl
Low-density lipoprotein	54 mg/dl	100-159 mg/dl
Very-low-density lipoprotein	18 mg/dl	0-40 mg/dl
HIV card test	Negative	
HBsAg	Negative	
HCV	Negative	

**Table 2:** CSF analysis done on the day of admission of the patient, along with their normal reference ranges. CSF: cerebrospinal fluid; units/L: units/liter; mg/dL: milligram/deciliter; IU/L: international units/liter; cumm: cubic millimeter

Laboratory parameter	Results	Normal values
Lactic dehydrogenase	71 units/L	<40 units/L
Protein-CSF	62 mg/dl	15-45 mg/dl
Glucose-CSF	65 mg/dl	50-80 mg/dl
pH	7.1	7.4
Adenosine deaminase	7.551 IU/L	15.7-21.3 IU/L
Total cells	21 cells/cumm	<5 cells/cumm

**Figure 2:**
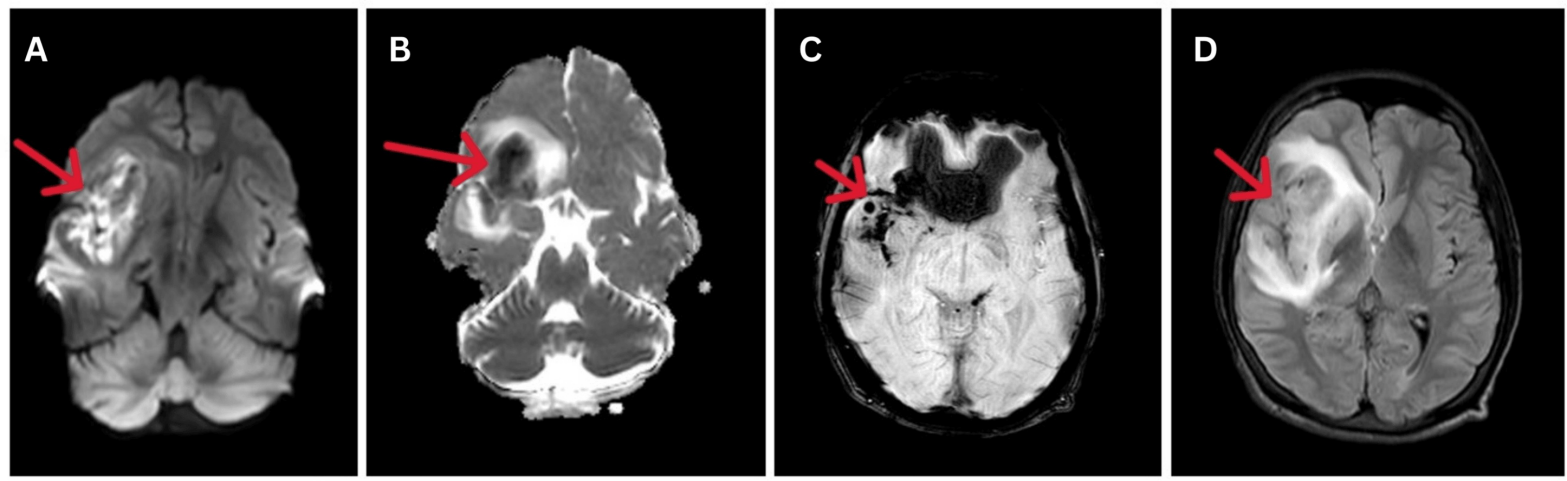
(A) Diffusion, (B) ADC, (C) SWI, and (D) FLAIR sequences of MRI of the brain suggestive of acute infarct with haemorrhagic transformation in the right temporal-frontal lobes (red arrows). ADC: apparent diffusion coefficient; SWI: susceptibility-weighted imaging; FLAIR: fluid-attenuated inversion recovery; MRI: magnetic resonance imaging

A 2D echocardiography was done during the course of hospitalization which was suggestive of normal contractile heart chambers and showed good blood stream along the valves. Regular electrocardiography was done to monitor any abnormal heart rhythms. The patient was diagnosed with viral encephalitis and was started on injection of acyclovir 500 mg eight hourly, injection of dexamethasone 8 mg eight hourly, injection of mannitol 100 ml eight hourly, and other supportive treatment. He gradually responded to treatment and got relief from the symptoms in 10 days. Due to some personal reasons, the patient was discharged against medical advice after 12 days of hospital stay. He was discharged with a Grade 2 Modified Rankin Score (mRS). Unfortunately, the patient returned to the hospital after 15 days of discharge with complaints of an initial encounter. Magnetic resonance imaging (MRI) of the brain suggested acute infarction with haemorrhagic transformation in the right frontal-parietal-temporal regions involving the right ganglion-capsular region and right corona radiata. Diffuse right-sided cerebral oedema was also noted (Figure 2). The patient was immediately shifted to the medicine intensive care unit for further evaluation and management.

Repeat lumbar puncture was planned, and CSF analysis was suggestive of findings as shown in Table 3. Anti-cyclic citrullinated peptide (CCP) antibodies, p-antineutrophil cytoplasmic antibodies (ANCA), c-ANCA, anti-*Saccharomyces cerevisiae* antibodies (ASCA), and anti-Sjögren syndrome-related antigen A (SSA) were done. Out of these, only the anti-SSA antibody returned positive; thus, the SS diagnosis was confirmed.

**Table 3:** CSF analysis done on the second admission of the patient, along with their normal reference ranges. CSF: cerebrospinal fluid; units/L: units/liter; mg/dL: milligram/deciliter; IU/L: international units/liter; cumm: cubic millimeter

Laboratory parameter	Results	Normal values
Lactic dehydrogenase	85 units/L	<40 units/L
Protein-CSF	72 mg/dl	15-45 mg/dl
Glucose-CSF	68 mg/dl	50-80 mg/dl
pH	7.25	7.4
Adenosine deaminase	11.24 IU/L	15.7-21.3 IU/L
Total cells	7 cells/cumm	<5 cells/cumm

**Figure 3:**
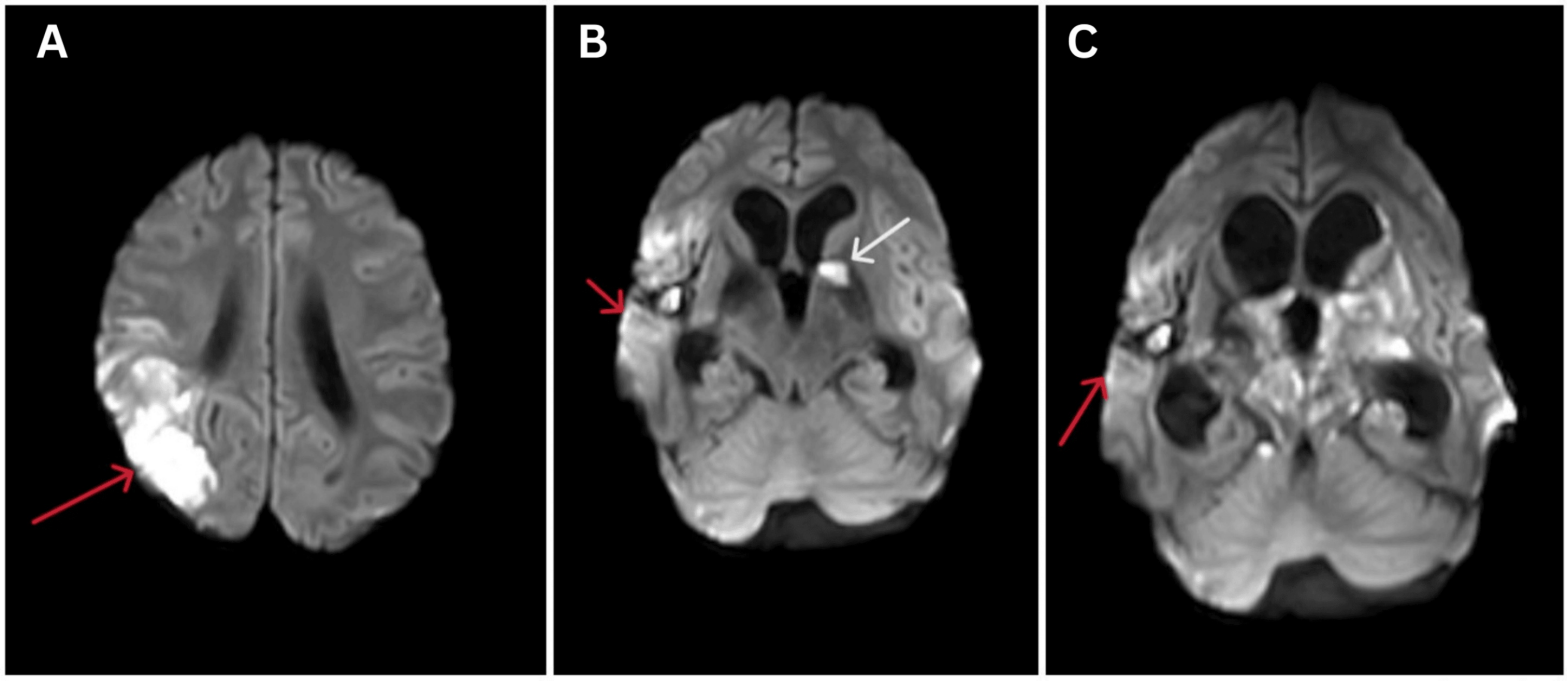
Serial MRI of the brain s/o acute infarcts (A) in the right parietal lobe (red arrow), (B) in the head of the left caudate nucleus, globus pallidus, and left internal capsule (red arrow) with periventricular ooze (white arrow), and (C) in the left lentiform, medial hypothalami, and midbrain (red arrow). MRI: magnetic resonance imaging

The patient was started on intravenous methylprednisolone 1 gm once a day for five days and monitored closely. On the fifth day of steroid therapy, his condition deteriorated, Glasgow Coma Scale (GCS) dropped, and the patient was intubated and taken on mechanical ventilation. Again, an MRI of the brain screening was done after five days, suggesting a new acute infarct with dilatation of the ventricular system with periventricular ooze (Figure 3).

**Figure 4:**
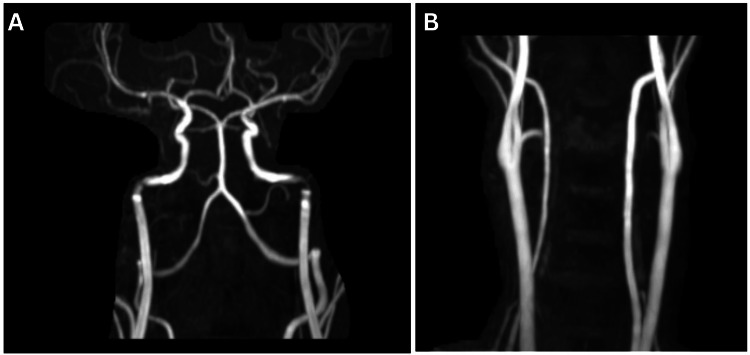
MR angiography showing (A) the internal carotid, vertebral arteries, and circle of Willis and its branches and (B) the arch of the aorta, bilateral common carotid, and external carotid appearing normal in course and calibre. MR: magnetic resonance

MR angiography was done, which suggested that the arch of the aorta, bilateral common carotid, external and internal carotid, vertebral arteries, and circle of Willis and its branches are normal in course and calibre (Figure 4). No obvious filling defect or abnormal dilatation was detected.

Immunosuppressants like cyclophosphamide were planned but could not be administered in time due to the rapidly deteriorating condition of the patient. Despite all the measures taken, the patient couldn't be revived and expired after one week of steroid therapy.

## Discussion

SS is a multisystem autoimmune disorder that presents a vast array of symptom clusters, including different systems or various metabolic and electrolyte abnormalities, which may even confuse clinicians as neurologic manifestations [4,5]. Many times, the clustering of various autoimmune disorders has been reported in the same patient [6]. Between 10% and 60% of SS patients experience neurological problems, which frequently manifest as peripheral neuropathy from small-vessel vasculitis [7]. In rare cases, there may be involvement of the CNS, presenting with symptoms such as transverse myelitis, meningoencephalitis, demyelinating disease, and mental disorders [8]. Acute ischemic strokes (AIS), which are caused mainly by accelerated atherosclerosis and vasculitis, are an even less common occurrence [9].

Studies using aortic distensibility, pulse wave velocity, and carotid intimal media thickness as surrogate indicators have demonstrated subclinical atherosclerosis in SS patients [9]. The significance of atherosclerosis is still up for debate, though. Primary SS patients did not have an elevated incidence of atherosclerosis-related AIS, according to statewide Taiwanese research. This finding has since been confirmed by a number of other investigations [10]. 

Nonetheless, there are a few case reports describing the development of AIS, which is thought to be predominantly caused by vasculitis [10-12]. According to single-centre Korean research, the overall prevalence of vasculitis in young stroke patients was about 2% [13]. Changes observed in the cerebral angiography were indicative of a more pronounced vasculitic pathophysiological process. Unfortunately, the lack of recommendations and data makes managing cerebral infarcts connected to vasculitis, such as SS, frequently challenging. Although there is currently insufficient proof, immunomodulation with corticosteroids, steroid-sparing medications (such as azathioprine and cyclophosphamide), and intravenous immunoglobulin treatment may be helpful. This is especially true for CNS involvement, which is far less common [14]. The results of trials involving biologic drugs, such as rituximab, are still up for debate, but most experts appear to agree that patients with severe diseases or complications should be the only ones who may use them [15].

## Conclusions

Despite advancements in therapy, CNS involvement in SS is a potential consequence that is still challenging to control. To the best of our knowledge, the literature has only documented one instance of SS presenting only with AIS as a result of large-vessel vasculitis. Even though it is uncommon, clinicians should be on the lookout for signs of SS as a possible cause of stroke, particularly in young patients.
